# Acute Sulcal FLAIR Hyperintensity in Severe Tick-Borne Encephalitis: A Potential Prognostic Marker

**DOI:** 10.3390/life15111655

**Published:** 2025-10-23

**Authors:** Vincent Böhm, Bogdan-Andrei Ianosi, Caterina Kulyk, Franz Gruber, Maria Lorenz, Thomas Mitterling, Amadeus Hauser, Stephan Eger, Ulrike Köhl, Serge Weis, Sibylle Wimmer, Michael Sonnberger, Raimund Helbok

**Affiliations:** 1 Department of Neurology, Johannes Kepler University Linz, 4020 Linz, Austria; 2 Clinical Research Institute for Neurosciences, Johannes Kepler University Linz, 4020 Linz, Austria; 3 Helios Klinikum Emil von Behring, 14165 Berlin, Germany; 4 Division of Neuropathology, Neuromed Campus, Department of Pathology and Molecular Pathology, Kepler University Hospital, Johannes Kepler University Linz, 4021 Linz, Austriaserge.weis@kepleruniklinikum.at (S.W.); 5 Department of Neuroradiology, Neuromed Campus, Kepler University Hospital, 4021 Linz, Austria

**Keywords:** TBE, infectious encephalitis, prognostic, marker, pathophysiology, diagnostics, sulcal hyperintensity

## Abstract

(1) Background: To report two cases of severe tick-borne encephalitis (TBE) in elderly patients presenting with a previously undescribed subarachnoid T2/FLAIR hyperintensity on repeated MRI examinations, which may serve as an early imaging biomarker of disease severity. (2) Methods: Two unvaccinated 82-year-old patients (one male and one female) presented with acute encephalitis and required intensive care. Serial brain MRI, EEG, CSF analysis, and neurophysiological assessments were performed. (3) Results: Both patients showed rapid progressive neurological deterioration in the context of TBE, confirmed by elevated serum and CSF IgM and IgG titers. Early follow-up MRI revealed striking sulcal hyperintensities on T2/FLAIR sequences, interpreted as protein-rich subarachnoid inflammatory changes. These changes paralleled clinical worsening and resolved on follow-up imaging. The male patient developed meningoencephalomyeloradiculitis, remained comatose, and died from respiratory failure (the brain and spinal cord were examined postmortem). The female patient had meningoencephaloradiculitis with severe dysphagia and was discharged with a modified Rankin Scale score of four. Both patients demonstrated epileptiform EEG activity. The CSF analysis revealed markedly elevated total protein, lactate, tau protein, and CXCL13, as evidence of blood–brain barrier disruption and inflammatory neurodegeneration. (4) Conclusions: We describe acute subarachnoid T2/FLAIR hyperintensity in TBE as an imaging feature that may correlate with severe systemic inflammation and a poor prognosis. This radiological finding could serve as a potential early prognostic marker in TBE.

## 1. Introduction

Tick-borne encephalitis (TBE) is an endemic infection in Central and Eastern Europe, caused by Flaviviruses, which are typically transmitted by Ixodes ticks. Climate change has expanded the vector’s range and prolonged seasonal activity, contributing to an increased incidence in many countries [1,2]. To date, no causal treatment exists, emphasizing the importance of prognostic markers to guide clinical decision-making [3,4]. While neuroimaging plays a key role in differential diagnoses, no radiographic predictors of disease severity have been established so far [5]. Here, we report a previously undescribed early imaging feature—acute subarachnoid T2/FLAIR hyperintensity—observed in two elderly patients with severe TBE, a finding that may have prognostic value.

## 2. Case Reports

### 2.1. Case 1

An 82-year-old previously independent man presented with high fever, somnolence, and left-sided hemiparesis following a biphasic disease course. Several tick bites were reported in the preceding four weeks; vaccination status for TBE was negative. Upon admission, he was somnolent, partially oriented, and had moderate left arm paresis.

**Figure 1:**
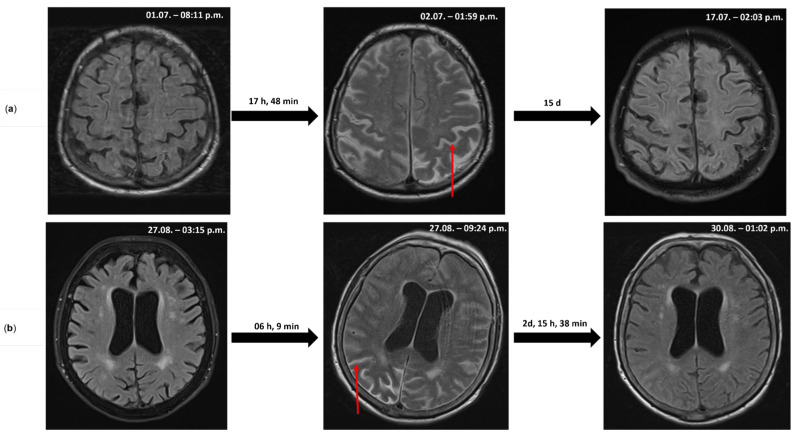
Serial cerebral MRI scans (T2/FLAIR): The initial MRI on admission (left) demonstrates normal CSF signal. Following rapid clinical deterioration, repeat imaging (middle) revealed acute sulcal hyperintensities (example: red arrow), likely representing proteinaceous, inflammatory CSF accumulation secondary to acute blood–brain barrier disruption. Follow-up imaging (right) shows resolution of these signal changes over time. (**a**) Case 1 and (**b**) Case 2. All MR images were acquired under standardized institutional protocols. Gadolinium contrast was administered during each MRI session; the displayed FLAIR/T2-weighted images were obtained prior to the subsequent contrast administration within the same examination. The exact date (day/month) and time of MRI sequence acquisition (hh:mm) are indicated in the upper right corner of each image. d, days; h, hours; and min, minutes.

Laboratory studies revealed leukocytosis (13.1 G/L) with minimal CRP elevation (1.3 mg/dL) (Table 1). CSF analysis showed pronounced pleocytosis (880 cells/µL), elevated lactate, total protein, and CXCL13 levels (Table 2). Initial MRI was unremarkable except for contrast enhancement of the meninges. Within 18 h, the patient rapidly deteriorated to a comatose state. Repeat MRI revealed pronounced sulcal hyperintensity on T2/FLAIR sequences (Figure 1a), interpreted as proteinaceous, inflammatory CSF. EEG confirmed nonconvulsive status epilepticus, treated with levetiracetam (40 mg/kg) and lacosamide (5.3 mg/kg). Due to lack of improvement in vigilance, tracheostomy was performed. Spinal MRI revealed cervical contrast enhancement and root involvement, consistent with meningoencephalomyeloradiculitis. NCS/EMG showed severe axonal damage in all limbs, as well as spontaneous activity suggestive of anterior horn involvement. TBE infection was confirmed by positive IgM/IgG titers in serum and CSF, without evidence of neuroborreliosis or other concomitant infections; however, he was not tested for West Nile virus infection. Despite cessation of epileptic activity and exclusion of other causes of impaired consciousness (delirium, electrolyte imbalance, etc.), the patient remained comatose with alpha–theta coma EEG activity. MRI on day 15 showed complete resolution of FLAIR hyperintensity. Flaccid paralysis and unreactive EEG without clinical improvement, and considering the patient’s age, led to the interdisciplinary decision to withhold therapy. The patient died 44 days after admission.

Postmortem analysis revealed diffuse cerebral edema and widespread nodular inflammatory infiltrates of cytotoxic T lymphocytes and activated microglia, with perivascular lymphocytic cuffing (Figure S1). Lesions were prominent in the frontobasal cortex, hippocampus, amygdala, basal ganglia, thalamus, brainstem, cerebellum, and spinal cord, including nerve roots and meninges.

### 2.2. Case 2

An 82-year-old woman without prior TBE vaccination reported a history of transient fever, headache, vomiting, and diarrhea. After a symptom-free interval of 8 days, she developed neck stiffness, dysarthria, mild left hemiparesis, and tremor with rigor of the right arm. The initial brain MRI was normal. Within a few hours the patient deteriorated to a comatose state (Glasgow coma scale 9), presenting with aggravated left-sided hemiparesis and tachycardia (new onset atrial fibrillation). Leukocyte count was 10.5 G/L, CRP 0.7 mg/dL (Table 1). Initial CSF analysis showed 41 cells/µL and elevated protein and lactate (Table 2). TBE infection was confirmed via CSF and serum antibody titers. West Nile virus infection was excluded by negative test results.

MRI within 6 h of deterioration revealed subarachnoid FLAIR hyperintensities (Figure 1b). EEG showed lateralized periodic discharges (LPDs) over the right frontal region. Levetiracetam (35 mg/kg) was initiated. Severe dysphagia required percutaneous endoscopic gastrostomy (PEG) placement.

Follow-up MRI after 2.5 days showed clear regression of subarachnoid signal changes without parenchymal lesions. After extended rehabilitation, the patient was discharged with residual left hemiparesis and dysphagia (mRS 4).

## 3. Discussion

We present two cases with T2/FLAIR-MRI subarachnoid hyperintensities in patients with confirmed TBE and rapid clinical deterioration. In both cases, this finding appeared abruptly in temporal association with clinical deterioration, supporting its potential utility as an early imaging marker of a severe inflammatory response and blood–brain barrier disruption.

The postmortem histology of Case 1 confirmed widespread neuroinflammation dominated by T-cells and microglial activation, consistent with virally induced injury. Of particular note, the extensive inflammatory involvement of the brainstem is likely to have been a decisive factor in the irreversible loss of brainstem functions observed clinically. Elevated CSF protein, tau, lactate, and CXCL13 levels (after excluding concomitant infections) in both patients are known to correlate with poor outcomes in TBE [4,6].

Although sulcal hyperintensity on T2/FLAIR-weighted imaging has not previously been reported in the context of TBE, this radiologic feature is recognized in other infectious and inflammatory disorders. Infectious etiologies include viral encephalitis such as West Nile virus (WNV) and the herpes virus, bacterial infections, and fungal pathogens including cryptococcal meningoencephalitis [7,8,9,10]. Case 1 was not tested for WNV infection; however, he did not show additional clinical features commonly associated with WNV, such as gastrointestinal symptoms or a maculopapular rash. Case 2 was tested for WNV, and the results were negative. In addition, non-infectious inflammatory conditions such as cerebral amyloid angiopathy-related inflammation and systemic lupus erythematosus have also been associated with sulcal hyperintensity [11,12].

Taken together, sulcal hyperintensity may reflect a final common pathophysiology of leptomeningeal inflammation and blood–brain barrier dysfunction and could serve as an early radiographic indicator for severe TBE. Further studies are needed to validate this observation in larger cohorts.

## 4. Limitations

Several limitations should be acknowledged. First, FLAIR sulcal hyperintensities have been described following repeated gadolinium-enhanced MRI examinations [13]. To address this confounder, we reviewed TBE patients in our institutional database who underwent repeated MRI within 48 h. None demonstrated comparable hyperintensities. In contrast, in one of our severely afflicted cases the abnormality was already present on the initial scan, making a solely contrast-related artifact unlikely. We therefore consider the finding biologically meaningful and consistent with severe blood–brain barrier disruption.

Second, sulcal hyperintensities have been attributed to hyperoxygenation, particularly in children under general anesthesia with high inspired oxygen concentrations [14,15]. In our patients, oxygen supplementation was limited to 2 L/min via nasal cannula, making hyperoxygenation an unlikely explanation.

Third, while our report suggests a novel radiographic correlate in TBE, sulcal hyperintensity on T2/FLAIR-weighted imaging is not specific to this condition. Similar findings have been reported for other infectious and inflammatory diseases [7,8,9,10,11,12]. In addition, other potential causes of sulcal FLAIR hyperintensity, such as seizures and ischemia, must also be considered [16,17]. Post-ictal leptomeningeal FLAIR abnormalities have been reported and can mimic infectious or inflammatory sulcal hyperintensity. They are most often focal and unilateral; are typically restricted to the epileptogenic hemisphere; and frequently occur together with cortical swelling, gyriform (cortical) FLAIR hyperintensity, transient diffusion abnormalities (DWI/ADC changes), and sometimes post-contrast leptomeningeal or gyral enhancement [18,19,20], none of which were observed in our patients. Additionally, there were no clinical signs of seizures at the time of the clinical deterioration, and the very short interval between the initial and follow-up MRI makes an epileptic etiology unlikely. Furthermore, diffusion-weighted imaging revealed no acute ischemic lesions, rendering cerebral ischemia an improbable explanation.

Finally, the absence of a control group precludes definitive conclusions. Our observations remain hypothesis-generating and require validation in larger cohorts.

## Figures and Tables

**Table 1:** Consecutive serology testing, (Laboratory Units) and [reference ranges].

Consecutive Serology Testing							
	**Case 1**				**Case 2**		
Days after onset of neurological symptoms	1	3	17		4	6	19
Days after presentation	1	3	17		2	4	17
Leukocytes (G/L) [4.4–9.4]	13.1				10.5		
Thrombocytes (G/L) [186–400]	235				157		
Hemoglobin (g/dL) [12.3–15.3]	14.1				12.9		
CRP (mg/dL) [0–0.5]	1.3				0.7		
Sodium (mmol/L) [136–145]	126				137		
Potassium (mmol/L) [3.5–4.5]	3.9				3.5		
GFR (mL/min/1.7) [70–130]	87				87		
CK (U/L) [0–170]	70				97		
LDH (U/L) [135–250]	140				259		
TBE IgM (Ratio) [0.80]	3.76	5.16	2.82		6.15	6.07	6.21
TBE IgG (RU/mL) [0–16]	16.4	18.53	165.51		48.08	132.79	>200

**Table 2:** Consecutive CSF testing, (Laboratory Units) and [reference ranges].

Consecutive CSF Testing								
	**Case 1**					**Case 2**		
Days after onset of neurological symptoms	1	3	17	36		4	6	19
Day after presentation	1	3	17	36		2	4	17
Leukocytes (/uL) [0–4]	880	120	15	11		41	74	44
Glucose-Ratio [0.5–0.7]	0.51	0.5	0.5	0.6		0.52	0.42	0.5
Lactate (mmol/L) [1–2.1]	3.7	4.15	2.61	2.14		3.1	3.2	3.17
Protein levels (mg/dL) [15–45]	121	147	136	137		151	123	98.8
CSF to serum albumin ratio (/1000) [0.50–10]	23.9		28.8			40.4	30.4	19.7
Tau Protein (pg/mL) [146–404]	480					801	876	
TBE IgM (L/S-Index) [0–1.3]	2.35	4.71	6.27			3	2	18.91
TBE IgG (L/S-Index) [0–1.3]	negative	2.7	0.72			1.89	4.22	1.84
FLCK (L/S) × 1000 [0–16.8]	128.14		297.49			366.22	696.43	
CXCL13 (pg/mL) [0–20]	<4	101.6	95.2				189.8	

## Data Availability

The original contributions presented in this study are included in the article/Supplementary Material. Further inquiries can be directed to the corresponding author.

## Supplementary Materials

The following supporting information can be downloaded at https://www.mdpi.com/article/10.3390/life15111655/s1: Figure S1: Immunohistochemical analysis demonstrates extensive inflammatory infiltration in the spinal cord and brain of Case 1.

## Abbreviations

The following abbreviations are used in this manuscript:

MRI	Magnetic resonance imaging
FLAIR	Fluid-attenuated inversion recovery
DWI	Diffusion-weighted imaging
ADC	Apparent diffusion coefficient
TBE	Tick-borne encephalitis
CSF	Cerebrospinal fluid
EEG	Electroencephalography
LPDs	Lateralized periodic discharges
NCS	Nerve conduction study
PEG	Percutaneous endoscopic gastrostomy
mRS	Modified Rankin scale
GFR	Glomerular filtration rate
CK	Creatine kinase
LDH	Lactate dehydrogenase
FLCK	Free light chains kappa
CXCL13	C-X-C motif chemokine 13
WNV	West Nile virus
