# Supplementary material for: Acute Sulcal FLAIR Hyperintensity in Severe Tick-Borne Encephalitis: A Potential Prognostic Marker
Source: Life (Basel). 2025 Oct 23;15(11):1655. doi: 10.3390/life15111655 (PMC12653168; doi:10.3390/life15111655)

Figure S1. Immunohistochemical analysis demonstrates extensive inflammatory infiltration in the spinal cord and brain of Case 1. (a) Spinal cord section with diffuse HLA-DR positivity indicating microglial activation. **(b)** Same level stained for CD8, demonstrating cytotoxic T-lymphocyte infiltration (red arrow). **(c), (d)** Hematoxylin–eosin (HE) staining and **(e)** CD8 positivity, show perivascular lymphocytic infiltrates (red arrow), together with diffuse **(f), (g)** infiltration in the brainstem. Similar findings are seen in the hippocampus **(h, i)**. **(j)** Widespread microglial activation with **(k)** microglial nodule formation is demonstrated by HLA-DR positivity, exemplified in the hippocampus. Magnifications: **(a)** 4×, **(b)** 5×, **(c, f, h, j)** 10×, **(d, e, i, k)** 40×, **(g)** 60x. Red rectangle marks corresponding region displayed at higher magnification.

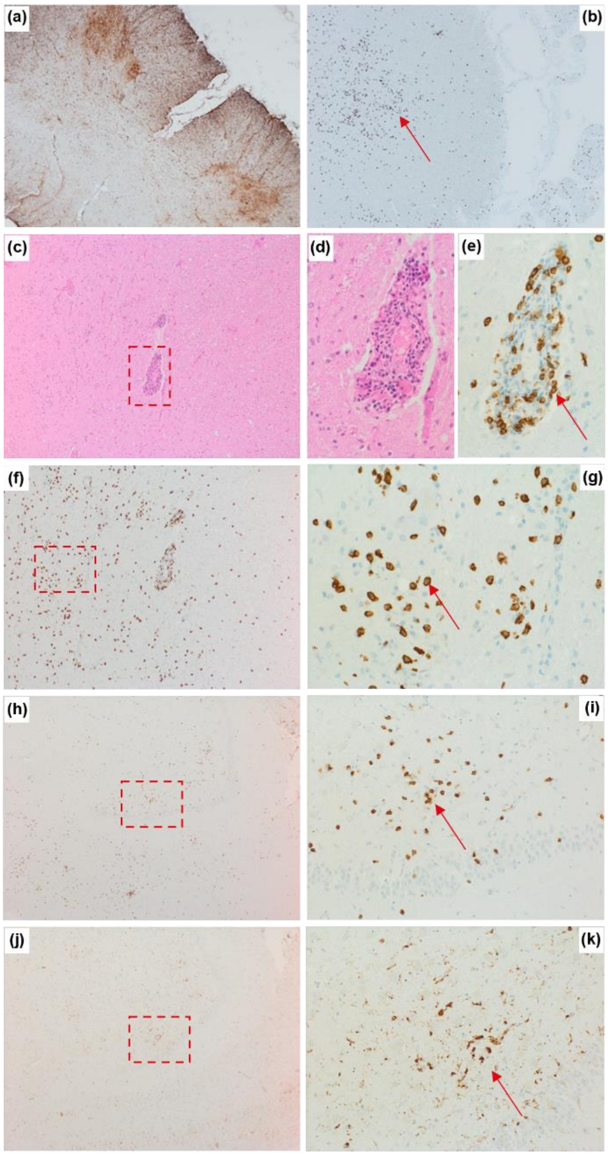

Supplement: Supplementary file 1 [file life-15-01655-s001.zip › life-3924383-supplementary.pdf]
